# An Adaptive Deep Learning Framework for Multi-Label Chest X-Ray Diagnosis Using a Hybrid CNN–Transformer Architecture and Class-Wise Ensemble Fusion

**DOI:** 10.3390/diagnostics16081227

**Published:** 2026-04-20

**Authors:** Chi-Feng Hsieh, Hsu-Hsia Peng, Yu-Hsiang Tsai, Chia-Ching Chang, Cheng-Hsuan Juan, Hsian-He Hsu, Chun-Jung Juan

**Affiliations:** 1 Department of Biomedical Engineering and Environmental Sciences, National Tsing Hua University, Hsinchu 300, Taiwan; 2 Department of Medical Imaging, China Medical University Hsinchu Hospital, Hsinchu 302, Taiwan; 3 Department of Management Science, National Yang Ming Chiao Tung University, Hsinchu 300, Taiwan; 4 Department of Medical Imaging, China Medical University Hospital, Taichung 404, Taiwan; 5 Graduate Institute of Biomedical Electronics and Bioinformatics, National Taiwan University, Taipei 106, Taiwan; 6 Department of Radiology, Tri-Service General Hospital and National Defense Medical University, Taipei 114, Taiwan; 7 Department of Radiology, School of Medicine, College of Medicine, China Medical University, Taichung 406, Taiwan; 8 Department of Computer Science and Information Engineering, National Taiwan University, Taipei 106, Taiwan

**Keywords:** chest radiography, deep learning, hybrid CNN–transformer, multi-label classification, thoracic disease

## Abstract

**Background/Objectives**: To develop and externally evaluate a deep learning framework for multi-label thoracic disease classification on chest radiographs using hybrid convolutional neural network (CNN)–transformer architectures, hierarchical scalar-weighted fusion, and ensemble strategies. **Methods**: This retrospective, multi-center study utilized publicly available datasets: NIH ChestX-ray14 (112,120 images; 30,805 patients) for model development and internal testing, and CheXpert (223,415 images) plus ChestX-Det10 (3578 images) for external validation. Nine CNN–transformer hybrids were systematically benchmarked, and the proposed model incorporated multi-scale DenseNet121 features, scalar-weighted fusion, positional encodings, and cross-attention. Four post hoc ensemble methods were explored, including a class-wise Top-3 Grid Search. Performance was evaluated using AUROC as the primary metric, along with precision, recall, F1-score, accuracy, specificity, positive predictive value, and negative predictive value. Statistical comparisons were performed using bootstrapped resampling and appropriate parametric or non-parametic tests. **Results**: On the NIH internal test set, the proposed hybrid model achieved a mean AUROC of 0.8495, which was significantly higher than that of the DenseNet121 baseline (0.8441, *p* = 0.032). The Top-3 Grid Search ensemble further improved internal performance, achieving a mean AUROC of 0.8577 (*p* < 0.00001). On external validation, the ensemble consistently outperformed DenseNet121, achieving mean AUROCs of 0.6500 on CheXpert (*p* < 0.001) and 0.6592 on ChestX-Det10 (*p* < 0.001). Per-class analysis revealed significant improvements for clinically important conditions such as cardiomegaly, mass, and pneumothorax. Grad-CAM visualizations demonstrated the strong alignment of predicted abnormalities with radiologically relevant regions. **Conclusions**: This CNN–transformer framework, particularly when combined with class-wise ensemble strategies, provided modest but statistically significant improvements in multi-label chest X-ray classification. External validation suggested partial generalizability across datasets, although performance remained moderate under domain shift.

## 1. Introduction

Chest radiography (CXR) remains one of the most widely utilized imaging modalities in clinical practice owing to its accessibility, rapid acquisition, and diagnostic versatility [1]. However, increasing imaging volumes and persistent shortages of radiologists contribute to diagnostic delays, inter-reader variability, and overlooked abnormalities [2,3,4]. These challenges are particularly evident in high-throughput environments such as emergency departments and under-resourced healthcare systems.

Artificial intelligence (AI) has emerged as a promising adjunct in radiological workflows, with recent studies demonstrating its potential as a second reader. For instance, Topff et al. showed that AI-assisted double reading enabled the detection of clinically relevant missed findings, including lung nodules, pneumothoraces, and consolidations, in routine reporting [5]. Likewise, Schalekamp et al. reported that AI could safely exclude up to 53% of normal CXRs at a conservative threshold, reducing radiologist workload by 15% while maintaining a 98% negative predictive value for urgent findings [6].

Despite these advances, most prior deep learning approaches rely on conventional convolutional neural networks (CNNs) [7,8,9,10,11] or adopt transformer-based modules [12,13,14] in relatively straightforward manners. These strategies often underuse complementary local and global feature representations, which are critical for capturing fine-grained lesion details and broader contextual patterns. To address this limitation, we systematically designed and evaluated nine CNN–transformer hybrid architectures incorporating diverse fusion strategies, including sequential and parallel integration, scalar-weighted multi-stage feature fusion, and cross-attention mechanisms.

The aim of this study is not to replicate clinical triage systems or to propose workflow replacement, but rather to advance AI-based diagnostic modeling by improving multi-label thoracic disease classification. We prioritized sensitivity to minimize the risk of missed abnormalities, thereby aligning with the concept of AI-assisted double reading. This emphasis is particularly relevant in settings where radiologist expertise is limited, and where AI tools may potentially serve as preliminary screening or second-reader support systems to flag suspicious cases for closer review [5,15].

We therefore hypothesized that hybrid architectures integrating local lesion detection with global contextual reasoning would enhance diagnostic performance compared with standard CNN baselines. To test this, we trained and validated our models on the large-scale NIH ChestX-ray14 dataset and assessed external generalizability using two independent datasets (CheXpert and ChestX-Det10) without additional fine-tuning by systematically benchmarking multiple architectural strategies. This study aims to provide insights into the design principles that may improve robustness, sensitivity, and clinical relevance in AI-assisted chest radiograph interpretation.

## 2. Materials and Methods

This retrospective, multi-center study utilized three publicly available chest radiograph (CXR) datasets: NIH ChestX-ray14 (*n* = 112,110; USA) [16], CheXpert (*n* = 224,316; USA) [17], and ChestX-Det10 (*n* = 3533; China) [18]. All the datasets used in this study were publicly available and fully de-identified, and no identifiable personal information was accessible to the authors. No interaction or intervention with human subjects was involved. According to the Declaration of Helsinki, 45 CFR 46, and the official government notices issued by the Department of Health, Executive Yuan, Taiwan—Scope of Human Research Cases Exempt from Review by the Research Ethics Committee and Scope of Human Research Cases Exempt from Obtaining the Research Subject’s Consent [19,20]—this type of study is exempt from ethics committee review and from the requirement for informed consent. Therefore, this study was exempt from obtaining ethical approval at the present institution. The NIH ChestX-ray14 dataset was used for model development and internal testing, whereas CheXpert and ChestX-Det10 served as external test sets to evaluate cross-institutional generalizability.

**Figure 1:**
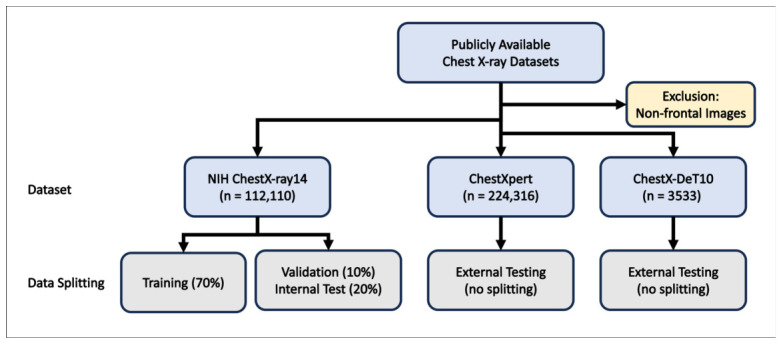
Patient flow diagram of dataset selection and allocation. Blue boxes represent dataset cohorts (e.g., NIH ChestX-ray14, ChestXpert, ChestX-Det10); light yellow boxes represent exclusion steps; Gray boxes represent data splitting or evaluation protocols.

The overall dataset allocation is illustrated in Figure 1. After excluding non-frontal images, 112,110 CXRs from NIH ChestX-ray14, 224,316 from CheXpert, and 3533 from ChestX-Det10 were included. The NIH dataset was split on a patient-wise basis into training (70%), validation (10%), and internal test (20%) sets. CheXpert and ChestX-Det10 were used exclusively for external testing.

**Figure 2:**
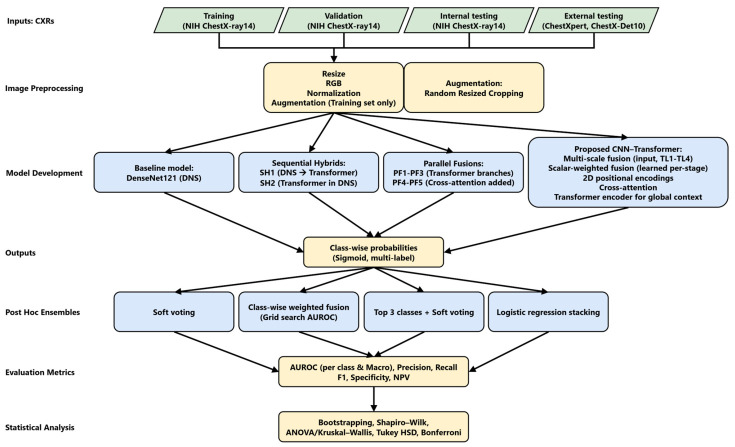
Study pipeline. Workflow includes dataset preparation, image preprocessing, model development (DenseNet121 baseline, hybrid CNN–transformer variants, and the proposed model), ensemble strategies, performance evaluation, and statistical analysis.

The study workflow is depicted in Figure 2. The input images were preprocessed by resizing them to 224 × 224 pixels, converting them to RGB, and applying ImageNet normalization. Random resized cropping was applied only to the training set for data augmentation. We then constructed multiple model architectures, including a DenseNet121 baseline, two sequential hybrids, five parallel fusion variants, and the proposed CNN–transformer model. Each model generated class-wise sigmoid probabilities, which were further combined through four post hoc ensemble strategies, including soft voting, class-wise weighted fusion, top 3 soft voting, and logistic regression stacking. Model performance was evaluated on the internal test set and validated externally on the CheXpert and ChestX-Det10 datasets using AUROC as the primary metric, along with threshold-dependent measures.

**Figure 3:**
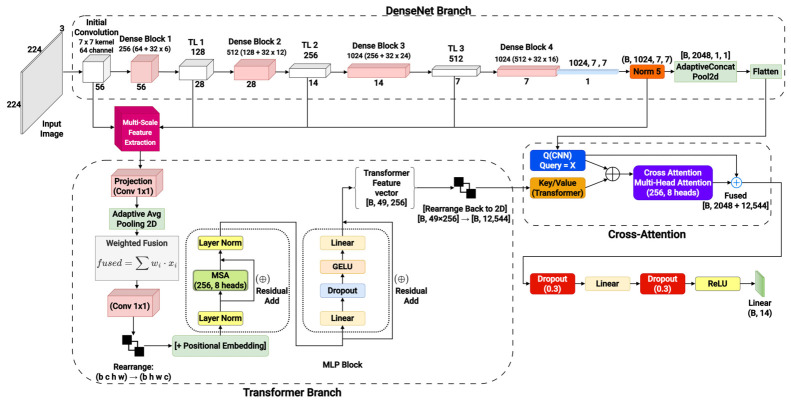
Schematic overview of the proposed convolutional neural network (CNN)–transformer hybrid architecture for multi-label chest X-ray classification. Input radiographs are processed in parallel through a DenseNet backbone and a transformer encoder branch. Multi-scale features are integrated via scalar-weighted fusion and refined using cross-attention, enabling the model to capture both localized and global contextual information. Final outputs are class-wise sigmoid predictions for thoracic diseases.

The architectural details of the comparative models are provided in Supplementary S1. A densely connected CNN (DenseNet121, DNS) was used as the baseline (Figure S1). Eight hybrid CNN–transformer models were developed: two sequential hybrids—Sequential Hybrid V1 (SH1), appending a transformer module after DNS (Figure S2), and Sequential Hybrid V2 (SH2), inserting the transformer module before Dense Block 3 (Figure S3)—and six parallel fusion variants (PF1–PF6) with independent transformer branches or cross-attention modules (Figure 3 and Figures S4–S8). These variants enabled the systematic evaluation of fusion timing, feature encoding, and attention mechanisms.

The proposed CNN–transformer architecture (Figure 3) integrates DenseNet121 [21] with a lightweight transformer encoder [22]. Six feature maps, extracted from the input and transition layers TL1–TL4, were projected into a shared 256-dimensional space using 1 × 1 convolutions and downsampled to 7 × 7 resolution. Adaptive scalar-weighted fusion with learned stage-wise coefficients dynamically balanced semantic contributions across feature hierarchies [23]. The fused tensor was tokenized, enriched with 2D sinusoidal positional encodings, and processed by transformer encoder layers for global contextual reasoning. A CNN-derived global vector was aligned with transformer outputs through cross-attention to refine representation learning. Mathematical formulations are provided in Supplementary S2.

To exploit complementary model strengths, four post hoc ensemble strategies were applied. Soft voting averaged sigmoid predictions across all models. Class-wise weighted fusion optimized per-class weights by grid search to maximize AUROC. Logistic regression stacking trained a meta-classifier on concatenated model outputs to capture non-linear prediction combinations. Top-3 Grid Search ensemble selected the three best-performing models per-class based on validation AUROC, combined via soft voting. All ensemble strategies were evaluated on NIH, CheXpert, and ChestX-Det10 datasets without additional fine-tuning.

The primary metric was the area under the receiver operating characteristic curve (AUROC), reported per-class and as a macro-average [24]. Threshold-dependent metrics included precision, recall (sensitivity), F1-score, accuracy, specificity, and negative predictive value (NPV) [25]. For multi-label classification, each disease class was treated as an independent binary prediction task, and class-specific probability thresholds were determined using Youden’s Index [26] on the validation set to balance sensitivity and specificity. These fixed thresholds were then applied to the corresponding test set predictions to convert sigmoid probabilities into binary labels. Notably, AUROC was used as the primary metric because it is threshold-independent, whereas the threshold-based metrics were used to evaluate classification performance at the selected operating points. Formal definitions of all metrics are provided in Supplementary S3.

The models were trained on the NIH ChestX-ray14 using patient-wise splits: 70% training, 10% validation, and 20% internal testing. DenseNet backbones were initialized with ImageNet weights, whereas transformer modules were randomly initialized. Training employed AdamW (learning rate = 1 × 10^−4^, weight decay = 1 × 10^−4^, batch size = 32). The hybrid models used a two-phase learning rate schedule: 5-epoch warm-up (GradualWarmupScheduler) followed by cosine annealing with warm restarts (T_0_ = 10, T_mult_ = 2, η_min_ = 1 × 10^−6^) [27]. DenseNet used standard cosine annealing. Training continued for up to 100 epochs, saving checkpoints when validation loss improved. A class-balanced binary cross-entropy loss [28], weighted by class frequencies, mitigated imbalance. For ensemble evaluation, models were trained independently and fused post hoc. Interpretability was assessed using Grad-CAM and attention visualizations. All experiments were implemented in PyTorch (v1.12) on an NVIDIA RTX 4090 GPU (24 GB VRAM).

Statistical analyses were conducted using Scikit-learn (v1.0), SciPy (v1.9), and Statsmodels (v0.13). Variability and statistical significance were assessed using a non-parametric bootstrapping (100 resamples per-class) for AUROC and recall distributions. Normality was tested with the Shapiro–Wilk test [29]. For normally distributed data, one-way ANOVA [30] was used to compare models; otherwise, the Kruskal–Wallis test [31] was applied. Significant group differences were further analyzed with Tukey’s HSD [32] or paired t-tests. Bonferroni correction was applied for multiple comparisons. A *p*-value of less than 0.05 was considered statistically significant.

## 3. Results

### 3.1. Dataset Characteristics

The NIH ChestX-ray14 dataset comprised 30,805 patients and 112,110 frontal-view chest radiographs. Following the predefined patient-wise split, 78,800 images were allocated for training, 11,212 for validation, and 22,108 for internal testing. External validation was performed using CheXpert (224,316 radiographs from 65,240 patients, USA) and ChestX-Det10 (3533 radiographs from a distinct clinical cohort in China). Demographic distributions included balanced sex representation and broad age ranges (18–90 years). Pathological prevalence varied across datasets: for example, cardiomegaly was present in 2.5% of NIH cases, while infiltration was observed in 17%. These heterogeneous patient populations provided a rigorous setting for assessing model robustness and cross-institutional generalizability.

### 3.2. Evaluation on the Internal Test Set

#### 3.2.1. Performance of Individual Models

Table S1 presents the overall diagnostic performance of nine individual models on the NIH ChestX-ray14 test set. Among these models, the proposed PF6 model achieved the highest mean AUROC, at 0.8495 (95% confidence interval [CI], 0.7130–0.9495). Its performance was significantly higher than that of DenseNet121 (0.8441; 95% CI, 0.8412–0.8470) and those of the other hybrid models (*p* < 0.05), with the exception of PF3, for which the difference was not statistically significant (*p* = 0.209).

#### 3.2.2. Ensemble Strategies

Comparisons of four post hoc ensemble strategies with the DenseNet121 baseline and the proposed hybrid CNN–transformer on the NIH ChestX-ray14 test set are presented in Table 1. All the ensemble approaches significantly outperformed both DenseNet121 and PF6 (all *p* < 0.01). Best per-class model selection achieved a mean AUROC of 0.851, significantly higher than PF6 (*p* < 0.01). AUROC-based weighted fusion (0.853) and logistic regression stacking (0.857) achieved further gains, each significantly superior to PF6 (*p* < 0.001). The Top-3 Grid Search ensemble achieved the highest overall performance with a mean AUROC of 0.858 (95% CI, 0.719–0.966), statistically superior to all other strategies (*p* < 0.001). These results demonstrate that post hoc ensemble learning consistently leveraged complementary model strengths to improve robustness.

#### 3.2.3. Per-Class Performance on Internal Test Set

**Figure 4:**
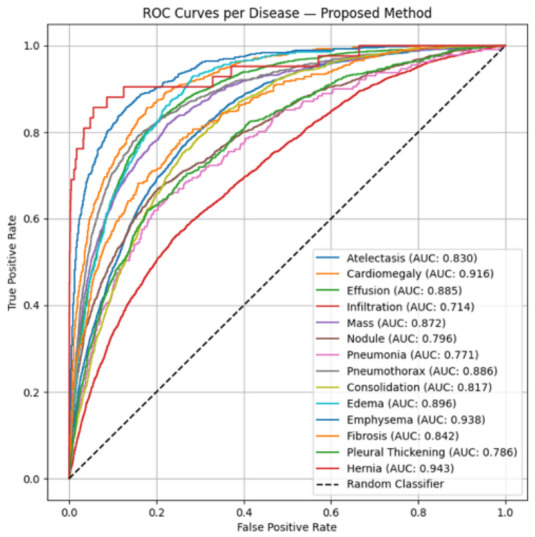
The receiver operating characteristic (ROC) curves of the proposed hybrid model for 14 thoracic disease classes on the NIH ChestX-ray14 test set. AUROC values ranged from 0.714 for infiltration to 0.943 for hernia. The highest AUROC values were observed for hernia (0.943), emphysema (0.938), and cardiomegaly (0.916), indicating strong discriminative performance for these disease classes.

The ROC curves for the 14 thoracic disease classes predicted by the proposed model are shown in Figure 4. The AUROC values ranged from 0.714 for infiltration to 0.943 for hernia, indicating variable but generally good discriminative performance across disease categories.

**Figure 5:**
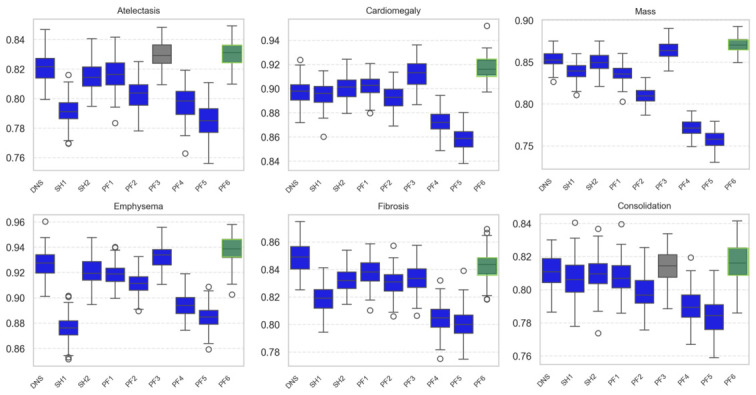
Bootstrapped AUROC distributions for six representative thoracic disease classes across the nine individual models on the NIH ChestX-ray14 test set. Each boxplot represents 100 bootstrap resamples. For readability, only six representative classes are shown here. The proposed model (PF6) is shown at the far right of each panel (green box). Box colors indicate the statistical significance of the comparison between PF6 and DenseNet121, with blue representing *p* < 0.0001 and gray representing *p* ≥ 0.05. Circles represent outliers, defined as values lying beyond 1.5× the interquartile range (IQR).

To further evaluate the effect of architectural enhancements, we compared bootstrapped per-class AUROC distributions across model variants. For readability, six representative disease classes are shown (Figure 5), while the complete results for all 14 classes are provided in Supplementary Figure S9. In these representative classes, the proposed model (PF6) generally outperformed DenseNet121, particularly for cardiomegaly, emphysema, and mass.

Table 2 presents a per-class AUROC comparison between the proposed model and the DenseNet baseline. The proposed method significantly outperformed the DenseNet baseline in atelectasis, cardiomegaly, consolidation, effusion, emphysema, infiltration, mass, and pneumothorax, but underperformed in fibrosis and pleural thickening.

Table 3 presents a per-class AUROC comparison between the Top-3 ensemble strategy and the DenseNet baseline. The Top-3 ensemble significantly improved AUROC across all 14 thoracic disease classes relative to DenseNet121 (all *p* < 0.001). The largest gains were observed in pneumonia (from 0.7558 to 0.7855; ΔAUROC = 0.0297), mass (from 0.8532 to 0.8734; ΔAUROC = 0.0202), and nodule (from 0.7888 to 0.8045; ΔAUROC = 0.0158). Clinically important conditions also showed marked improvement, including cardiomegaly (from 0.9056 to 0.9186; ΔAUROC = 0.0129), hernia (from 0.9455 to 0.9605; ΔAUROC = 0.0150), fibrosis (from 0.8395 to 0.8515; ΔAUROC = 0.0120), and pleural thickening (from 0.7818 to 0.7976; ΔAUROC = 0.0157). Even for infiltration, which had a relatively low baseline AUROC, the ensemble improved performance from 0.7073 to 0.7212 (ΔAUROC = 0.0139; *p* < 0.001).

#### 3.2.4. Failure Analysis: Proposed Model vs. Ensemble Strategy

To further elucidate the strengths and limitations of the proposed hybrid model, we performed a per-class comparison with the Top-3 Grid Search ensemble across multiple diagnostic metrics (Table 4).

The ensemble achieved an overall higher recall (0.79) than the proposed method (0.77) at the expense of lower accuracy (0.78 vs. 0.79) and specificity (0.79 vs. 0.80). Specifically, the ensemble demonstrated statistically significant improvements in sensitivity for atelectasis, infiltration, nodule, pneumothorax, and pleural thickening (all *p* < 0.001), as well as modest improvements for consolidation (*p* < 0.01) and hernia (*p* < 0.05). These gains were accompanied by consistent reductions in specificity and accuracy. In contrast, cardiomegaly, edema, and fibrosis showed the opposite trend: the proposed model achieved higher sensitivity (0.86, 0.93, and 0.78, respectively), whereas the ensemble achieved higher accuracy and specificity (0.850/0.850, 0.788/0.786, and 0.798/0.799, respectively). This finding highlights a class-specific trade-off between the proposed model and the Top-3 Grid ensemble strategy.

### 3.3. External Validation

#### 3.3.1. Per-Class Comparison Across Models and External Datasets

Cross-dataset validation was performed to assess the generalizability of the proposed framework (Table 5). To assess cross-domain generalizability, five models with internal-test AUROC values higher than 0.84 were selected, including DenseNet121, PF6, PF2, SH2, and PF1. Additionally, the Top-3 ensemble using soft voting, where model fusion weights were optimized via grid search, was added for comparison. Per-class and mean AUROC scores were computed using 100-trial bootstrapping.

In CheXpert, the Top-3 ensemble achieved a mean AUROC of 0.650, significantly higher than the DenseNet121 baseline (0.6318, *p* < 0.001). The largest improvements were observed in pneumothorax (from 0.5974 to 0.6366; ΔAUROC = 0.0392), effusion (from 0.4419 to 0.4799; ΔAUROC = 0.0380), and infiltration (from 0.7736 to 0.7950; ΔAUROC = 0.0214). Performance gains were also evident for pneumonia, from 0.6194 to 0.6403 (ΔAUROC = 0.0209), despite differences in labeling protocols between datasets. In ChestX-Det10, the Top-3 ensemble achieved a mean AUROC of 0.6592, compared with 0.6476 for DenseNet121 (*p* < 0.001). Substantial improvements were seen for atelectasis (from 0.6461 to 0.6862; ΔAUROC = 0.0401), consolidation (from 0.5862 to 0.6118; ΔAUROC = 0.0256), and mass (from 0.6994 to 0.7214; ΔAUROC = 0.0221). In contrast, performance decreased for nodule, from 0.6532 to 0.6174 (ΔAUROC = −0.0358; *p* < 0.001), likely reflecting variations in annotation quality and class prevalence. For emphysema, performance differences were not statistically significant (*p* = 0.407). Together, these results indicate that ensemble methods improved performance relative to the DenseNet121 baseline on both external datasets, although the absolute external AUROC values remained moderate and generalizability varied across disease classes.

#### 3.3.2. Model Interpretability via Grad-CAM

**Figure 6:**
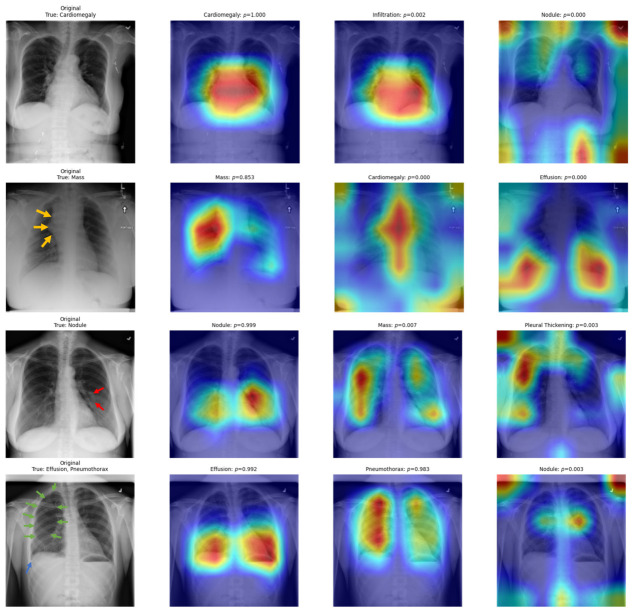
Gradient-weighted Class Activation Mapping (Grad-CAM) visualizations from the proposed hybrid model on representative cases from the NIH ChestX-ray14 dataset. Each row presents the original chest radiograph (left) followed by Grad-CAM heatmaps for the top predicted disease classes. The warmer regions (red/yellow) indicate stronger model attention, generally aligning with disease-relevant anatomical areas. The visual markers include: (1) yellow arrows indicating suspected mass regions, (2) red arrows marking pulmonary nodules, (3) blue arrow highlighting pleural effusion, and (4) green arrows identifying pneumothorax locations.

Representative Gradient-weighted Class Activation Mapping (Grad-CAM) visualizations are displayed in Figure 6, providing insight into the spatial regions contributing to model predictions. In correctly classified cases of cardiomegaly, pulmonary mass and nodule, the heatmaps demonstrated strong correspondence with clinically relevant anatomical regions, thereby supporting the interpretability of predictions. In addition, the heatmaps showed consistent localization in cases with dual disease classes, including pleural effusion and pneumothorax, further highlighting the model’s ability to capture coexisting pathologies. However, occasional diffuse or ambiguous activations were observed, suggesting that interpretability should be contextualized with clinical expertise to avoid potential misinterpretation.

## 4. Discussion

This study presents a systematically evaluated deep learning framework for multi-label chest X-ray classification based on hybrid CNN–transformer architectures and ensemble strategies. Rather than introducing a fundamentally new model family, the main contribution of this work lies in the structured comparison of multiple integration designs, including sequential and parallel fusion strategies, as well as post hoc ensemble methods, under both internal and external evaluation settings. The results suggest that hierarchical multi-stage fusion and class-wise ensemble selection can provide measurable performance gains over a DenseNet121 baseline.

The main contributions of this study are threefold. First, unlike previous works that primarily coupled CNNs and transformers in a linear fashion [33,34,35], we systematically explored multiple integration strategies and demonstrated that hierarchical multi-scale fusion with cross-attention achieves superior performance. Second, the use of scalar-weighted stage-wise fusion enabled the adaptive balancing of shallow and deep features, thereby improving sensitivity to subtle findings such as consolidation and infiltration. Third, we extended beyond single-model prediction by incorporating post hoc ensemble strategies. The Top-3 Grid Search ensemble achieved the best performance, reaching a mean AUROC of 0.8577 on the NIH internal test set, which was significantly higher than both the DenseNet121 baseline (0.8441; *p* < 0.001) and PF6 (0.8495; *p* < 0.001). On external validation, the Top-3 ensemble achieved statistically significant improvements over the DenseNet121 baseline on both CheXpert and ChestX-Det10. However, the absolute external AUROC values remained moderate (0.6500 and 0.6592, respectively), indicating that cross-dataset transferability was only partial. These findings suggest that, although the proposed framework improved robustness relative to the baseline, substantial domain shift remained across datasets. Possible contributors include differences in label definitions, annotation procedures, disease prevalence, image acquisition protocols, and population characteristics.

When benchmarked against prior methods, our Top-3 ensemble surpassed CNN-only architectures such as Thorax-Net (AUROC = 0.787) [36], DenseNet121 (0.79) [37], DCXNet (0.82) [38], CheXNet (0.8413) [8], CheXNet-PFN (0.846) [39], and CONVFCMAE (0.8523) [7], all of which rely primarily on localized feature extraction. Its performance was also comparable to Z-Net (0.858) [40], which represents one of the strongest CNN-based designs. Beyond CNNs, the ensemble outperformed transformer-only approaches, including DeiT-B (0.78) [37], SwinCheX (0.810) [41], and the Vision Transformer (0.837) [14], which emphasize global context modeling but often underperform in fine-grained lesion detection. These results support the potential value of combining local texture-sensitive CNN features with transformer-based global contextual modeling for chest X-ray classification. Furthermore, our ensemble exceeded other reported hybrid frameworks such as SA-DenseNet (0.8357) [33] and SynthEnsemble (0.854) [34], although it remained slightly below LungMaxVit (0.932) [35].

Unlike many prior studies restricted to internal evaluation, our framework was externally tested on two independent datasets, providing evidence of relative robustness across datasets, although the absolute external performance remained moderate. From a clinical perspective, the hybrid framework showed class-specific performance gains in several clinically important conditions, such as cardiomegaly, emphysema, mass, and pneumothorax. Ensemble strategies significantly improved sensitivity for atelectasis, infiltration, and pleural thickening, whereas the proposed model preserved relatively higher accuracy and specificity for some classes, including cardiomegaly and edema. These results demonstrate statistically significant but quantitatively modest improvements over the baseline and suggest that hybrid fusion and ensemble strategies may improve model robustness. However, the moderate external AUROC values indicate that further refinement and prospective validation are still required before clinical deployment can be considered.

Furthermore, interpretability remains a critical prerequisite for clinical translation. Our Grad-CAM visualizations demonstrated that the proposed framework consistently highlighted disease-relevant anatomical regions, thereby improving the technical interpretability of the model outputs. Nevertheless, mislocalized or ambiguous activations suggest that these attention maps should be interpreted cautiously and regarded as supportive visual aids rather than definitive evidence of clinical reasoning.

Nonetheless, several limitations warrant acknowledgment. First, persistent variability in categories such as infiltration and pleural thickening reflects labeling noise and overlapping radiographic features. Confidence calibration or expert-in-the-loop refinement may help address this issue. Second, our study focused on frontal-view CXRs, limiting applicability to lateral projections; future work should incorporate multi-view training. Third, clinical and demographic metadata (e.g., age, sex, or comorbidities) were not included, although such information could improve personalization and disease priors. Finally, the current model was optimized for research purposes; real-time deployment will require computational refinements such as quantization, pruning, or model distillation.

Future research should extend toward multimodal frameworks that integrate imaging with clinical metadata, laboratory tests, and longitudinal imaging. Federated learning approaches could further enhance generalizability while preserving data privacy. Most critically, prospective clinical trials are needed to evaluate workflow integration, interpretability, and radiologist–AI collaboration in real-world practice.

## 5. Conclusions

In conclusion, hybrid CNN–transformer architectures combined with multi-scale fusion and ensemble strategies provided modest but statistically significant improvements in multi-label chest X-ray classification compared with a DenseNet121 baseline. The external validation results suggest partial generalizability across datasets, although performance remained moderate under domain shift. These findings support the methodological potential of hybrid and ensemble designs, while further robustness testing and prospective clinical validation are needed before real-world implementation.

## Figures and Tables

**Table 1:** The mean AUROC of ensemble strategies on the NIH ChestX-ray14 test set with statistical comparison against the DenseNet121 baseline and the proposed model.

Strategy	Mean AUROC (95% CI)	*p*-Value vs. DenseNet121	*p*-Value vs. Proposed Method
DenseNet121 (Baseline)	0.8441 [0.8412, 0.8470]	–	***
Proposed Method (PF6)	0.8495 [0.7130, 0.9495]	***	–
Best Model Per-Class	0.8512 [0.7138, 0.9591]	***	**
AUROC-Based Weighted Fusion	0.8530 [0.7170, 0.9636]	***	***
Stacking (Logistic Regression)	0.8567 [0.7192, 0.9709]	***	***
Top-3 Grid Search Fusion	0.8577 [0.7186, 0.9664]	***	***

Note: Values represent mean AUROC with 95% confidence intervals derived from 100 bootstrap resamples. *p*-values were obtained using two-tailed paired *t*-tests comparing bootstrapped AUROC distributions. Significance thresholds: *p* < 0.01 (**), *p* < 0.001 (***).

**Table 2:** Per-class AUROC comparison between the DenseNet121 baseline and the proposed hybrid CNN–transformer model on the NIH ChestX-ray14 test set.

Disease Class	AUROC (DenseNet)	AUROC (Proposed)	∆AUROC (Proposed − DenseNet)	*p*-Value
Atelectasis	0.8215	0.8292	0.0078	***
Cardiomegaly	0.8957	0.9145	0.0189	***
Consolidation	0.8110	0.8162	0.0052	***
Edema	0.8956	0.8955	0.0001	0.9576
Effusion	0.8814	0.8847	0.0110	**
Emphysema	0.9265	0.9389	0.0079	***
Fibrosis	0.8488	0.8404	−0.0084	***
Hernia	0.9468	0.9442	−0.0026	0.0663
Infiltration	0.7050	0.7142	0.0092	***
Mass	0.8501	0.8713	0.0213	***
Nodule	0.7924	0.7954	0.0030	0.0598
Pleural Thickening	0.7889	0.7871	−0.0018	0.2155
Pneumonia	0.7688	0.7699	0.0011	0.4069
Pneumothorax	0.8754	0.8869	0.0115	***

Note: ∆AUROC = AUROC (Proposed) − AUROC (DenseNet). Significance levels: ** *p* < 0.01, *** *p* < 0.001. Exact *p* values are reported for non-significant differences.

**Table 3:** Per-class AUROC comparison between DenseNet121 baseline and the Top-3 Grid Search ensemble on the NIH ChestX-ray14 test set.

Disease Class	AUROC (DenseNet)	AUROC (Top-3 Grid)	∆AUROC (Top-3 Grid − DenseNet)	*p*-Value
Atelectasis	0.8288	0.8360	0.0072	***
Cardiomegaly	0.9056	0.9186	0.0129	***
Consolidation	0.8123	0.8217	0.0094	***
Edema	0.8937	0.9035	0.0098	***
Effusion	0.8823	0.8891	0.0068	***
Emphysema	0.9316	0.9434	0.0118	***
Fibrosis	0.8395	0.8515	0.0120	***
Hernia	0.9455	0.9605	0.0150	***
Infiltration	0.7073	0.7212	0.0139	***
Mass	0.8532	0.8734	0.0202	***
Nodule	0.7888	0.8045	0.0158	***
Pleural Thickening	0.7818	0.7976	0.0157	***
Pneumonia	0.7558	0.7855	0.0297	***
Pneumothorax	0.8841	0.8944	0.0103	***

Note: ∆AUROC = AUROC (Top-3 Grid Ensemble) − AUROC (DenseNet121). *p*-values were derived from Tukey’s HSD test applied to bootstrapped AUROC distributions (*n* = 100). Significance thresholds: *p* < 0.001 (***).

**Table 4:** Comparison of diagnostic performance metrics between the proposed hybrid CNN–transformer model and the Top-3 Grid Search ensemble across 14 disease classes on the NIH ChestX-ray14 test set, using Youden’s thresholds for binary classification.

Disease Class	Proposed Model						Top-3 Ensemble Strategy						*p*-Value
	PPV	Sensitivity	F1	Accuracy	Specificity	NPV	PPV	Sensitivity	F1	Accuracy	Specificity	NPV	
Atelectasis	0.26	0.78	0.39	0.7336	0.7279	0.9648	0.24	0.84	0.38	0.6983	0.6812	0.9724	***
Cardiomegaly	0.11	0.86	0.20	0.8225	0.8216	0.9954	0.13	0.83	0.22	0.8496	0.8501	0.9948	**
Effusion	0.38	0.81	0.52	0.8146	0.8150	0.9687	0.38	0.81	0.52	0.8140	0.8143	0.9687	0.914
Infiltration	0.33	0.56	0.41	0.7200	0.7536	0.8899	0.29	0.66	0.40	0.6565	0.6552	0.9012	***
Mass	0.16	0.82	0.27	0.7774	0.7752	0.9877	0.16	0.82	0.27	0.7767	0.7745	0.9877	0.862
Nodule	0.17	0.67	0.27	0.7893	0.7969	0.9744	0.16	0.70	0.27	0.7707	0.7750	0.9763	***
Pneumonia	0.03	0.66	0.06	0.7698	0.7710	0.9952	0.04	0.62	0.07	0.8204	0.8226	0.9950	0.102
Pneumothorax	0.20	0.79	0.32	0.8387	0.8411	0.9876	0.20	0.82	0.32	0.8302	0.8305	0.9893	***
Consolidation	0.11	0.76	0.20	0.7338	0.7326	0.9856	0.11	0.78	0.20	0.7285	0.7261	0.9868	**
Edema	0.06	0.93	0.11	0.7204	0.7164	0.9982	0.07	0.90	0.13	0.7880	0.7860	0.9975	**
Emphysema	0.13	0.85	0.23	0.8709	0.8712	0.9961	0.16	0.85	0.27	0.8966	0.8977	0.9961	0.532
Fibrosis	0.05	0.78	0.09	0.7474	0.7469	0.9952	0.06	0.75	0.11	0.7979	0.7988	0.9948	*
Pleural Thickening	0.10	0.62	0.18	0.8138	0.8203	0.9846	0.09	0.71	0.16	0.7527	0.7542	0.9872	***
Hernia	0.03	0.86	0.05	0.9443	0.9445	0.9997	0.01	1.00	0.02	0.7880	0.7876	1.0000	*
Average	0.15	0.77	0.24	0.7920	0.7957	0.9811	0.15	0.79	0.24	0.7836	0.7851	0.9848	–

Note: Metrics include precision (PPV), sensitivity, F1-score, accuracy, specificity, and negative predictive value (NPV). Significance levels: * *p* < 0.05, ** *p* < 0.01, *** *p* < 0.001.

**Table 5:** Per-class and mean AUROC scores on external datasets (CheXpert and ChestX-Det10) for DenseNet121 baseline, hybrid models, and the Top-3 Grid Search ensemble.

Dataset	Disease Class	DenseNet121	Proposed PF6	PF2	SH2	PF1	Top-3 Ensemble	∆AUROC	*p*-Value
**CheXpert**	Atelectasis	0.5489	0.5637	0.5677	0.4529	0.4809	0.5640	+0.0151	***
	Cardiomegaly	0.6822	0.6633	0.6557	0.5332	0.6258	0.6850	+0.0028	***
	Effusion	0.4419	0.4895	0.4910	0.5604	0.5003	0.4799	+0.0380	***
	Infiltration	0.7736	0.7845	0.7840	0.5601	0.6002	0.7950	+0.0214	***
	Pneumonia	0.6194	0.6110	0.6104	0.4128	0.4396	0.6403	+0.0209	***
	Pneumothorax	0.5974	0.5847	0.5716	0.5112	0.5312	0.6366	+0.0392	***
	Consolidation	0.7617	0.7731	0.7757	0.4387	0.6015	0.7802	+0.0185	***
	Edema	0.6520	0.6452	0.6387	0.5001	0.4905	0.6702	+0.0182	**
	Pleural Thickening	0.4716	0.4651	0.4741	0.4327	0.4624	0.4982	+0.0266	***
	Mean AUROC	0.6318	0.6292	0.6275	0.4702	0.5077	0.6500	+0.0182	***
**ChestX-Det10**	Atelectasis	0.6461	0.6433	0.6397	0.4385	0.4477	0.6862	+0.0401	***
	Consolidation	0.5862	0.6154	0.6154	0.4038	0.5414	0.6118	+0.0256	***
	Effusion	0.7093	0.7123	0.7156	0.4965	0.4897	0.7333	+0.0240	***
	Emphysema	0.8862	0.8792	0.8791	0.5155	0.5860	0.8905	+0.0043	0.4068
	Fibrosis	0.5823	0.5808	0.5815	0.5122	0.5734	0.5912	+0.0089	**
	Mass	0.6994	0.7192	0.7129	0.4949	0.4778	0.7214	+0.0221	***
	Nodule	0.6532	0.6103	0.6100	0.5032	0.4928	0.6174	−0.0358	***
	Pneumothorax	0.8833	0.8905	0.8951	0.4487	0.4367	0.8997	+0.0164	***
	Mean AUROC	0.6476	0.6466	0.6458	0.4801	0.5216	0.6592	+0.0116	***

Note: ∆AUROC = AUROC (Top-3 Ensemble)—AUROC (DenseNet121). *p*-values were derived from paired *t*-tests comparing bootstrapped AUROC distributions (*n* = 100) against the DenseNet121 baseline. Significance thresholds: *p* < 0.01 (**), *p* < 0.001 (***). Non-significant exact values are reported.

## Data Availability

The original contributions presented in this study are included in the article/Supplementary Material. Further inquiries can be directed to the corresponding authors.

## Supplementary Materials

The following supporting information can be downloaded at https://www.mdpi.com/article/10.3390/diagnostics16081227/s1, Figure S1: DenseNet-121 model; Figure S2: Sequential Hybrid V1 (SH1); Figure S3: Sequential Hybrid V2 (SH2); Figure S4: Parallel Fusion V1 (PF1); Figure S5: Parallel Fusion V2 (PF2); Figure S6: Parallel Fusion V3 (PF3); Figure S7: Parallel Fusion V4 (PF4); Figure S8: Parallel Fusion V5 (PF5); Figure S9: AUROC distributions per disease class for all model variants; Table S1: Mean AUROC comparison of model variants on the NIH ChestX-ray14 test set. Supplementary S1. Architectural Overview of Baseline and Comparative Models; Supplementary S2. Model Formulation and Attention Mechanism; Supplementary S3. Evaluation Metrics; Supplementary S4. Model-Wise Mean AUROC Comparison.

## Abbreviations

The following abbreviations are used in this manuscript:

AI	Artificial Intelligence
AUROC	Area Under the Receiver Operating Characteristic Curve
CNN	Convolution Neural Network
CXR	Chest X-ray
DNS	DenseNet121
NPV	Negative Predictive Value
PF1	Parallel Fusion V1 (Simple Fusion)
PF2	Parallel Fusion V2 (Cross Stage)
PF3	Parallel Fusion V3 (Cross Stage + PE + Weights)
PF4	Parallel Fusion V4 (Single Cross-Attention)
PF5	Parallel Fusion V5 (Double Cross-Attention)
PF6	Proposed Parallel Fusion V6
PPV	Positive Predictive Value (Precision)
SH1	Sequential Hybrid V1
SH2	Sequential Hybrid V2
