# Supplementary material for: An Adaptive Deep Learning Framework for Multi-Label Chest X-Ray Diagnosis Using a Hybrid CNN–Transformer Architecture and Class-Wise Ensemble Fusion
Source: Diagnostics (Basel). 2026 Apr 20;16(8):1227. doi: 10.3390/diagnostics16081227 (PMC13115109; doi:10.3390/diagnostics16081227)
Supplement: Supplementary file 1 [file diagnostics-16-01227-s001.zip › diagnostics-4226187-supplementary.pdf]

Supplementary Material

Supplementary S1. Architectural Overview of Baseline and Comparative Models

We provide detailed architectural descriptions and visualizations of the baseline DenseNet-121 backbone and the eight comparative fusion variants (SH1 to PF5) developed to evaluate the effectiveness of different CNN–Transformer integration strategies for chest X-ray classification. For clarity, all model diagrams are placed in this supplementary section with expanded captions defining abbreviations. The final proposed model (PF6) is presented and discussed in detail in the main manuscript.

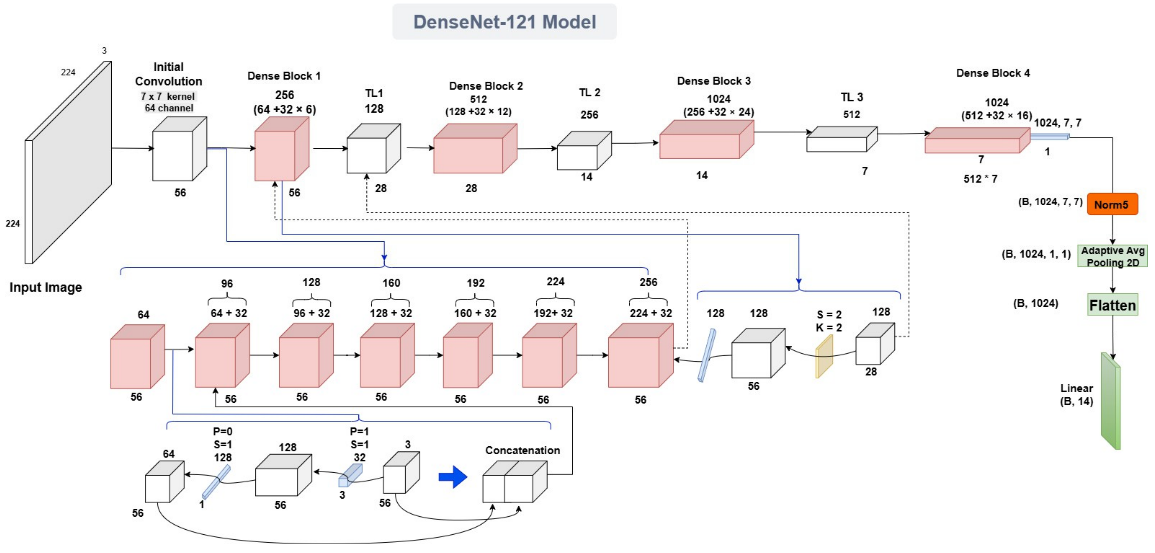

Figure S1: DenseNet-121 model: the architecture begins with a 7×7 convolution (stride 2, 64 channels), followed by four dense blocks (Dense Block 1–4) with growth rate *k*=32 and bottleneck layers (1×1 conv before 3×3 conv). Each dense block concatenates the outputs of all preceding layers along the channel dimension (blue arrows). Transition layers (TL1–TL3) consist of a 1×1 convolution and 2×2 average pooling for downsampling. Numbers above each block indicate output channels, while numbers

below indicate spatial resolution. After the final dense block, features are batch-normalized (Norm5), globally averaged, flattened, and passed to a linear layer for classification.

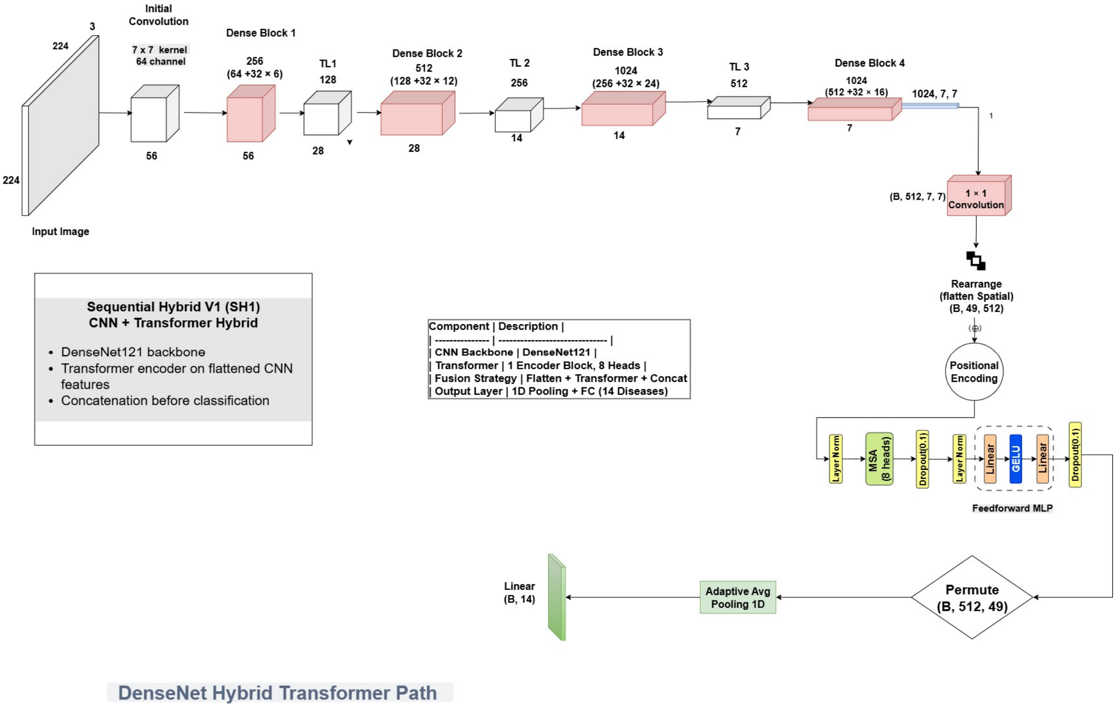

Figure S2: Sequential Hybrid V1 (SH1): DenseNet-121 features are flattened and passed to a transformer encoder with positional encodings, before concatenation with CNN features for classification. MSA = Multi-Head Self-Attention (8 heads), Norm = Layer Normalization, PE = Positional Encoding, FFN = Feedforward Network (Linear → GELU → Linear), FC = Fully Connected layer. Tensor dimensions are shown as (B, . . .), where B denotes batch size. Numbers above blocks indicate output channels or embedding dimensions; numbers below indicate spatial resolution.

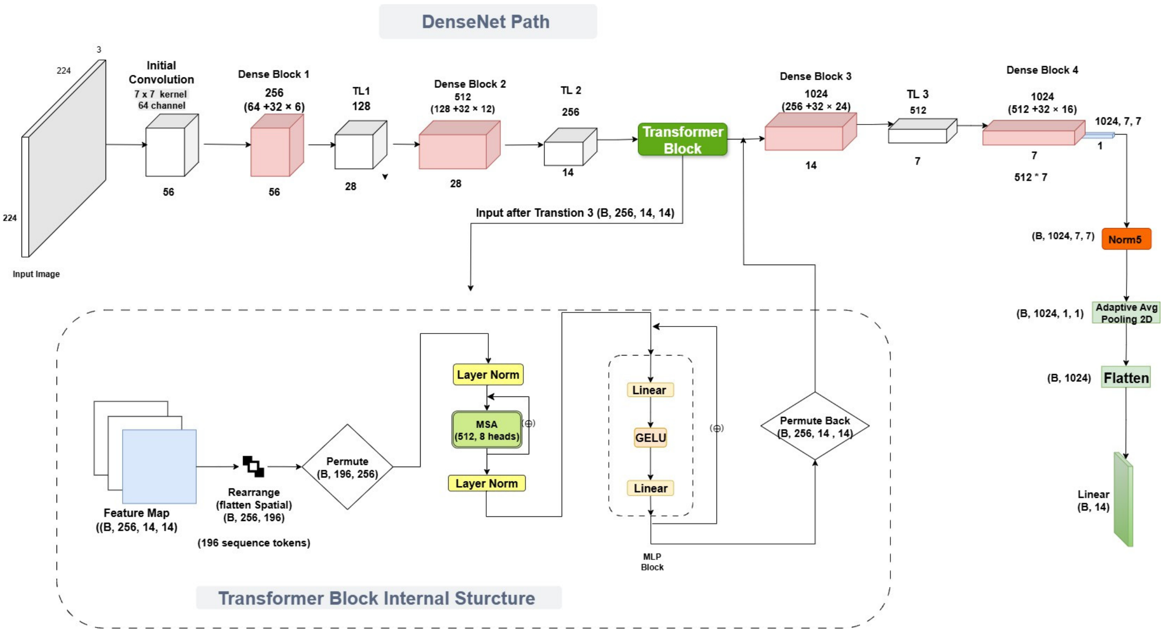

Figure S3: Sequential Hybrid V2 (SH2): Extends SH1 by injecting a transformer block into the DenseNet- 121 backbone between Transition Layer 2 (TL2) and Dense Block 3. The transformer block consists of LayerNorm, MSA = Multi-Head Self-Attention (8 heads), and an FFN = Feedforward Network (Linear → GELU → Linear) with Dropout for regularization. Norm = Layer Normalization, FC = Fully Connected layer. Tensor dimensions are denoted as (B, . . .), where B is the batch size. Numbers above blocks indicate output channels or embedding dimensions, and numbers below indicate spatial resolution.

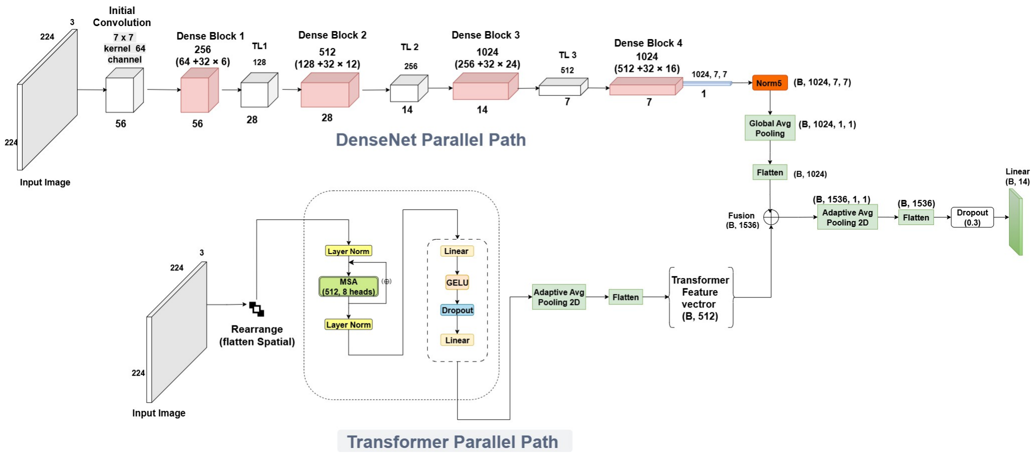

Figure S4: Parallel Fusion V1 (PF1): DenseNet-121 and a Transformer branch operate in parallel. The CNN path produces a feature vector after Dense Block 4 and global average pooling, while the Transformer path processes flattened image patches with positional encodings through LayerNorm, MSA = Multi-Head Self-Attention (8 heads), and an FFN = Feedforward Network (Linear → GELU → Dropout → Linear). Outputs from both branches are concatenated (Fusion) and passed to a fully connected (FC) layer for classification. Norm = Layer Normalization, GELU = Gaussian Error Linear Unit activation. Tensor dimensions are denoted as (B, . . .), where B is the batch size. Numbers above blocks indicate output channels or embedding dimensions, and numbers below indicate spatial resolution.

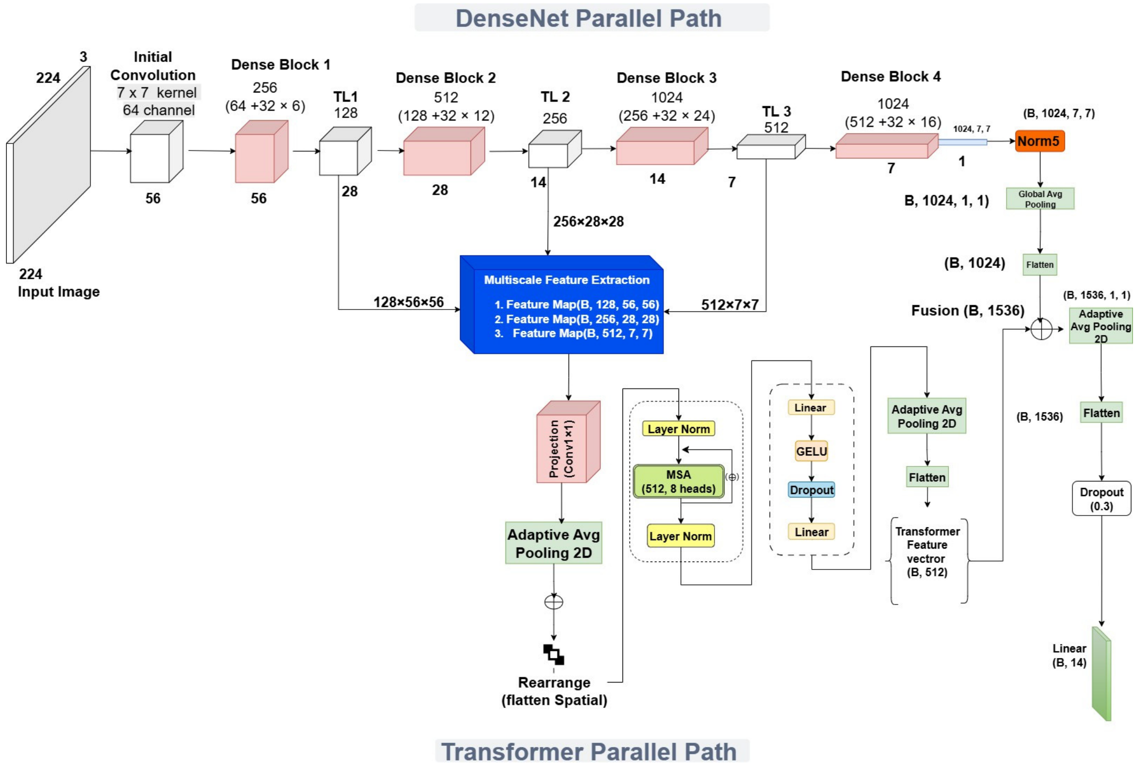

Figure S5: Parallel Fusion V2 (PF2): Unlike PF1, the transformer branch derives its tokens directly from multiscale CNN feature maps at different depths (e.g., after Dense Block 1, Transition Layer 2, and Dense Block 3), projected via a 1×1 convolution and pooled before rearrangement into sequences. The transformer block includes LayerNorm, MSA = Multi-Head Self-Attention (8 heads), and an FFN = Feedforward Network (Linear → GELU → Dropout → Linear). Outputs from the CNN and transformer branches are concatenated (Fusion) and passed through fully connected (FC) layers for classification. Norm = Layer Normalization, GELU = Gaussian Error Linear Unit activation. Tensor dimensions are denoted as (B, . . .), where B is the batch size. Numbers above blocks indicate output channels or embedding dimensions, and numbers below indicate spatial resolution.

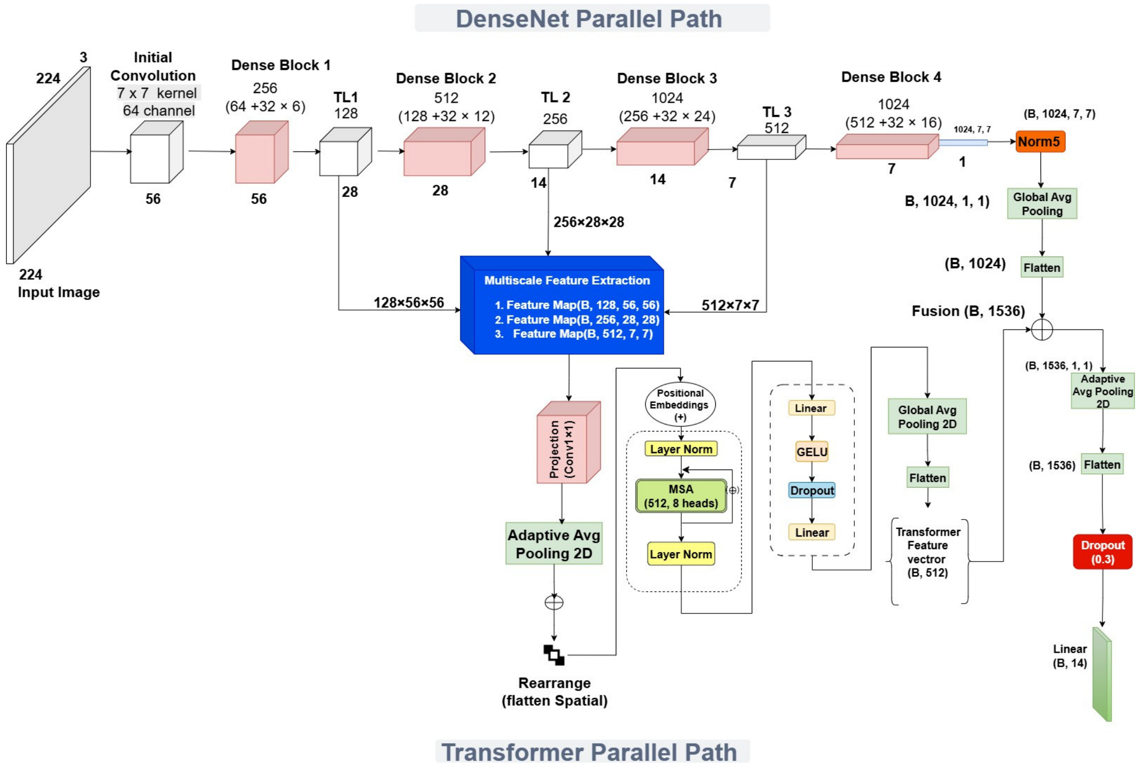

Figure S6: Parallel Fusion V3 (PF3): Extends PF2 by incorporating 2D positional encodings into the transformer branch to better preserve spatial relationships of CNN-derived features. Multiscale feature maps from different DenseNet stages are projected via a 1×1 convolution, pooled, and rearranged into sequences before being combined with positional embeddings. The transformer block includes LayerNorm, MSA quences before being combined with positional embeddings. The transformer block includes LayerNorm, MSA = Multi-Head Self-Attention (8 heads), and an FFN = Feedforward Network (Linear → GELU → Dropout → Linear). Outputs from the DenseNet and transformer branches are concatenated (Fusion) and passed to a fully connected (FC) classifier. Norm = Layer Normalization, GELU = Gaussian Error Linear Unit activation. Tensor dimensions are denoted as (B, . . .), where B is the batch size. Numbers above blocks indicate output channels or embedding dimensions, and numbers below indicate spatial resolution.

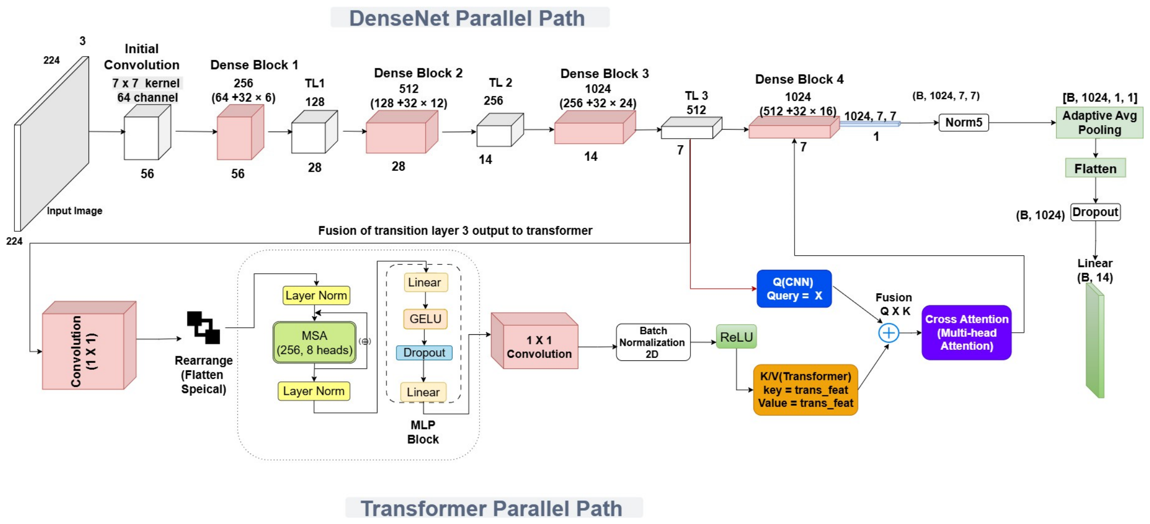

Figure S7: Parallel Fusion V4 (PF4): Enhances PF3 by introducing a cross-attention module that fuses CNN and transformer features after extraction. The DenseNet-121 branch produces high-level CNN features, while the transformer branch processes tokenized inputs with LayerNorm, MSA = Multi-Head Self-Attention (8 heads), and an FFN = Feedforward Network (Linear → GELU → Dropout → Linear). Cross-attention takes CNN features as the query (Q) and transformer features as key (K) and value (V), enabling local-global feature interaction before classification. Norm = Layer Normalization, GELU = Gaussian Error Linear Unit activation, FC = Fully Connected layer. Tensor dimensions are denoted as (B, . . .), where B is the batch size. Numbers above blocks indicate output channels or embedding dimensions, and numbers below indicate spatial resolution.

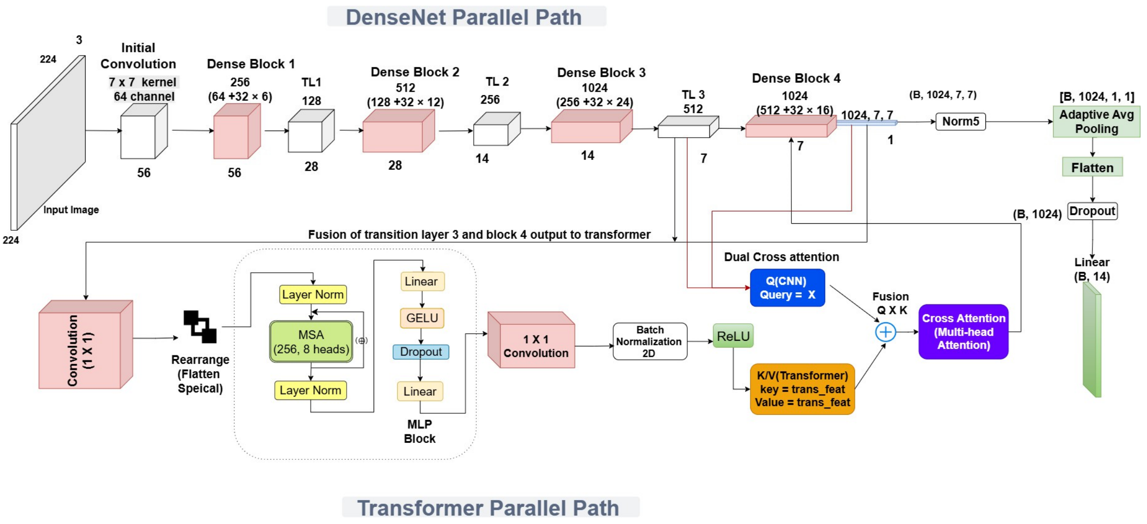

Figure S8: Parallel Fusion V5 (PF5): Extends PF4 by introducing dual cross-attention modules to en- hance CNN–transformer interaction. The first cross-attention block fuses CNN features from Transition Layer 3 (TL3) with transformer representations, while the second cross-attention block incorporates higher-level CNN features from Dense Block 4 (TL4 output). Queries (Q) are derived from CNN fea- tures, and Keys/Values (K/V) from transformer features, enabling deeper local-global feature alignment. The transformer block includes LayerNorm, MSA = Multi-Head Self-Attention (8 heads), and an FFN = Feedforward Network (Linear → GELU → Dropout → Linear). Norm = Layer Normalization, GELU = Gaussian Error Linear Unit activation, FC = Fully Connected layer. Tensor dimensions are denoted as (B, . . .), where B is the batch size. Numbers above blocks indicate output channels or embedding dimensions, and numbers below indicate spatial resolution.

Supplementary S2. Model Formulation and Attention Mechanism

Scalar-Weighted Feature Fusion. To enable adaptive feature combination, we implemented scalar- weighted fusion using learned coefficients:

$$F_{fused} = \sum_{i=1}^{6} \alpha_i \cdot P_i$$

(S1)

where P_i denotes the projected feature map from the *i*-th DenseNet stage. This formulation allows dynamic reweighting of shallow and deep semantic features [8, 1].

Transformer-Based Cross-Attention. The fused features are tokenized and passed to a transformer encoder with 2D sinusoidal positional encodings. A CNN-based global feature vector serves as the query in a cross-attention module that aligns local and global features via:

$$Attention(Q, K, V) = softmax(\frac{QK^T}{\sqrt{d}})V$$

(S2)

where Q, K, and V denote the query, key, and value matrices respectively, and d is the dimension of the keys. The final fused representation is used for 14-class multi-label prediction via a sigmoid-activated classification head [5].

Ensemble Fusion Formulation

In the class-wise grid search ensemble strategy, we computed the final prediction for each disease class c

using a weighted average over M models:

$$\widehat{y_c} = \sum_{i=1}^{M} \alpha_c^{(i)} \cdot y_c^{(i)}, \sum \alpha_c^{(i)} = 1$$

(S3)

where y_c^i denotes the predicted probability from model *i* for class *c*, and $\alpha_c^{(i)}$ is the optimized weight assigned to that model for class *c*.

Weights were grid-searched on the validation set to maximize AUROC per class, enforcing $\sum \alpha_c^{(i)} = 1$ *as a normalization constraint*

This strategy allowed the ensemble to emphasize the most reliable models per class, yielding higher average AUROC and better per-disease calibration.

Supplementary S3. Evaluation Metrics

We evaluated multi-label classification performance using both threshold-independent and threshold- based metrics. The primary evaluation metric was the area under the receiver operating characteristic curve (AUROC), which was computed on a per-class basis and then macro-averaged [2].

In addition to AUROC, we calculated the following threshold-based metrics:

$$Precision = \frac{TP}{TP+FP}$$

(S4)

$$Recall\ (Sensitivity) = \frac{TP}{TP+FN}$$

(S5)

$$F1\ score = \frac{2 \cdot Precision \cdot Recall}{recision+Recall}$$

(S6)

$$Accuracy = \frac{TP+TN}{TP+FP+PN+TN}$$

(S7)

$$Specificity = \frac{TN}{TN+FP}$$

(S8)

$$Negative\ predictive\ value\ (NPV) = \frac{TN}{TN+FNN}$$

(S9)

To determine the decision threshold for each disease class, Youden's Index [10], defined as the following equation, was calculated on the validation set. The resulting fixed thresholds were then applied to the NIH ChestX-ray14 test set and the external datasets (CheXpert and ChestX-Det10) for threshold-dependent evaluation, without any additional fine-tuning.

$$\text{Youden's Index} = \text{Sensitivity} + \text{Specificity} - 1 \tag{S10}$$

Supplementary S4. Model-Wise Mean AUROC Comparison

Table S1: Mean AUROC comparison of model variants on the NIH ChestX- ray14 test set.

**Model Variant**	**Mean AUROC**	**ΔAUROC**	***p* value**
DenseNet121 (DNS)	0.8441	+0.0054	*
Sequential Hybrid V1 (SH1)	0.8217	+0.0278	***
Sequential Hybrid V2 (SH2)	0.8410	+0.0085	**
Parallel Fusion V1 (PF1)	0.8407	+0.0088	*
Parallel Fusion V2 (PF2)	0.8279	+0.0215	***
Parallel Fusion V3 (PF3)	0.8424	+0.0070	0.2094
Parallel Fusion V4 (PF4)	0.8134	+0.0360	***
Parallel Fusion V5 (PF5)	0.8041	+0.0454	***
Proposed Parallel Fusion (PF6)	**0.8495**	–	–

Note: ΔAUROC is calculated as $\text{AUROC}_{\text{PF6}} - \text{AUROC}_{\text{Model}}$. Significance levels: * $p < 0.05$, ** $p < 0.01$, *** $p < 0.001$. Exact p-values are shown for non-significant comparisons ($p \geq 0.05$).

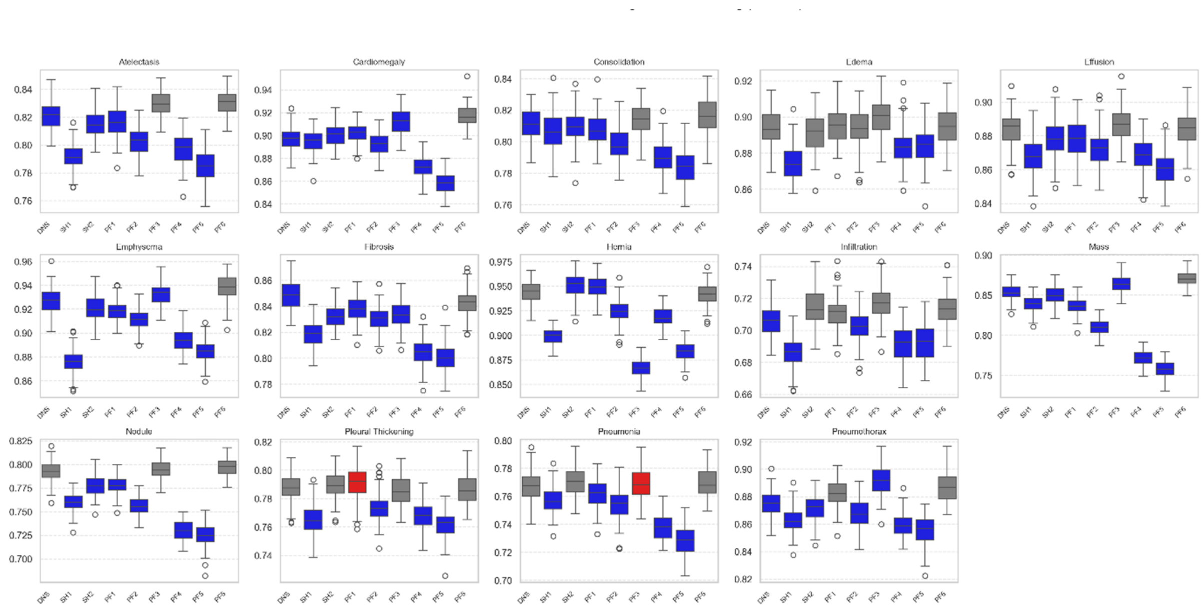

Figure S9. **Bootstrapped area under the receiver operating characteristic curve (AUROC) distributions per disease class for all model variants on the NIH ChestX-ray14 dataset.** Each boxplot represents the AUROC variability across 100 bootstrapped resamples. Statistically significant improvements of the proposed model compared with the DenseNet121 baseline are indicated. Box colors denote significance levels: blue (p < 0.0001), red (p < 0.001), green (p < 0.05), and gray (non-significant, $p \geq 0.05$).
